# Femtosecond Laser Thermal Accumulation-Triggered Micro-/Nanostructures with Patternable and Controllable Wettability Towards Liquid Manipulating

**DOI:** 10.1007/s40820-022-00840-6

**Published:** 2022-04-08

**Authors:** Kai Yin, Lingxiao Wang, Qinwen Deng, Qiaoqiao Huang, Jie Jiang, Guoqiang Li, Jun He

**Affiliations:** 1 Hunan Key Laboratory of Nanophotonics and Devices, School of Physics and Electronics, Central South University, Changsha, 410083 People’s Republic of China; 2 The State Key Laboratory of High Performance and Complex Manufacturing, College of Mechanical and Electrical Engineering, Central South University, Changsha, 410083 People’s Republic of China; 3 Key Laboratory of Testing Technology for Manufacturing Process of Ministry of Education, Southwest University of Science and Technology, Mianyang, 621010 People’s Republic of China

**Keywords:** Wettability, Femtosecond laser, Micro-/nanostructures, Thermal accumulation, Liquid manipulating

## Abstract

Versatile liquid manipulating surfaces combining patternable and controllable wettability have recently motivated considerable attention owing to their significant advantages in droplet-solid impacting behaviors, microdroplet self-removal, and liquid–liquid interface reaction applications. However, developing a facile and efficient method to fabricate these versatile surfaces remains an enormous challenge. In this paper, a strategy for the fabrication of liquid manipulating surfaces with patternable and controllable wettability on Polyimide (PI) film based on femtosecond laser thermal accumulation engineering is proposed. Because of its controllable micro-/nanostructures and chemical composition through adjusting the local thermal accumulation, the wettability of PI film can be tuned from superhydrophilicity (~ 3.6°) to superhydrophobicity (~ 151.6°). Furthermore, three diverse surfaces with patternable and heterogeneous wettability were constructed and various applications were successfully realized, including water transport, droplet arrays, and liquid wells. This work may provide a facile strategy for achieving patternable and controllable wettability efficiently and developing multifunctional liquid steering surfaces.

**Supplementary Information:**

The online version contains supplementary material available at 10.1007/s40820-022-00840-6.

## Highlights

- The patternable and controllable wettability via femtosecond laser thermal accumulation engineering is proposed for liquid manipulating.
- The wettability of polyimide film can be tuned from superhydrophilicity (~3.6°) to superhydrophobicity (151.6 °).
- Three diverse surfaces with patternable and heterogeneous wettability are constructed for application of water transport, droplet arrays, and liquid wells.

**Supplementary Information:**

The online version contains supplementary material available at 10.1007/s40820-022-00840-6.

## Graphical abstract

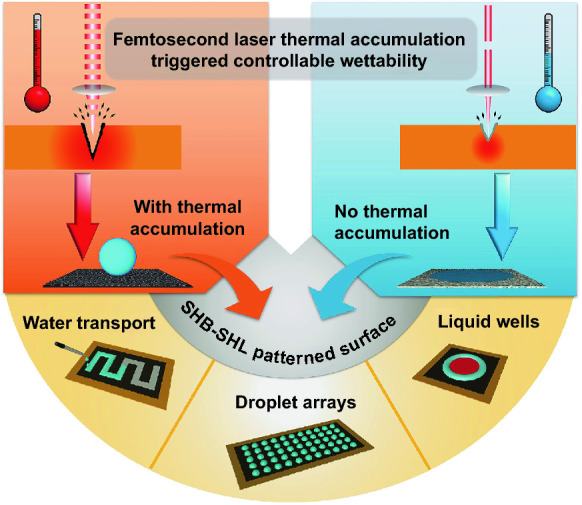

## Introduction

Surface wettability is critical to a range of practical applications [1–9], which is primarily governed by the surface micro-/nanostructures and chemical composition [10–12]. Versatile liquid manipulating surfaces with controllable microstructures and wettability have attracted considerable interest on account of their great potentials in scientific investigations, involving cellular screening [13, 14], droplet-solid impacting behavior [15, 16], liquid directional transport [17, 18], oil–water separation [19, 20], bubble assembling [21], and so forth. Currently, several methods have been explored to fabricate functional surfaces with superwettability, such as plasma treatment [22, 23], electrochemical-etching [24, 25], UV irradiation [26], and spray-coating [27, 28]. For instance, Li et al. [29] designed an adhesion-patterned surface on porous alumina plates by chemical vapor deposition and UV irradiation, which was successfully applied for controllable droplet rotational bouncing. Song et al. [30] developed a serial-wedge-shaped wettability pattern on an aluminum plate via electrochemical etching, Fluoroalkylsilane modification and laser scanning, which was competent for the realization of spontaneous and directional transportation of gas bubbles in an aqueous environment. However, there are still several deficiencies needed to be addressed, including complex treatments, high cost and uncontrollable wettability. Therefore, seeking a convenient and efficient way to fabricate multifunctional liquid manipulating surfaces with controllable wettability is urgently demanded.

Laser processing has emerged as an effective technique to control the wettability of solid surfaces, because different micro-/nanostructures can be directly prepared on various substrates by one-step laser scanning [31–33]. In most cases, the laser beam forms micro-/nanostructures on substrates for achieving desired wettability owing to the thermal effects, which is induced by laser ablation with high energy density. However, laser ablation with thermal effects is only serving for the fabrication of simply functional surfaces with non-tunable wettability, and it is hard to satisfy our up-to-date requirements. Compared with these conventional lasers, femtosecond laser has a variety of features, such as high energy intensity, high processing efficiency and environmental friendliness [34–37]. Nevertheless, almost all reported femtosecond laser fabrication technology has taken advantage of cold machining, which can only achieve one wettability without post-treatment [38, 39]. To our best knowledge, regulating the thermal accumulation of the femtosecond laser processing to achieve various patternable and controllable wettability for liquid manipulating has never been reported.

Herein, we put forward a femtosecond laser thermal accumulation engineering to prepare patternable and controllable wettability surfaces toward liquid manipulating on polyimide (PI) film. With the thermal accumulation developing, the PI film surface’s micro-/nanostructures and chemical composition can be modified accordingly, which leads to the continuously controllable wettability, from superhydrophilicity (~ 3.6°) to superhydrophobicity (~ 151.6°). Subsequently, a series of heterogeneous and patternable wettability surfaces are prepared and various liquid manipulating applications are successfully achieved, including water transport, droplet arrays and liquid wells.

## Experimental Section

### Femtosecond Laser Fabrication

In this work, PI film (∼0.1 mm) was selected as the raw material. PI film has exceptional advantages such as enduring high-low temperature resistance, good thermal stability, corrosion resistance, excellent mechanical properties, high electrical insulation, and radiation resistance. Additionally, PI film has low thermal conductivity and its melting temperature is ~ 280 °C. The laser beam (central wavelength of 1035 nm, pulse width of 350 fs) from a commercial femtosecond fiber laser system (HR-Femto-IR-50-40B, Huaray, China) was guided onto the sample surface and scanned along *x*–*y* directions through a two-mirror galvanometric scanner system (basiCube 10, Scanlab, Germany) with an F-Theta lens (focused length of 125 mm). The laser repetition rate, power, and scanning speed were used from 5 to 100 kHz, 80 to 900 mW, 50 to 250 mm s^−1^, respectively. The scanning spacing was fixed at 12 µm. All the treatments were carried out at room temperature.

### Characterization

A field emission scanning electron microscope (SEM, MIRA3 LMU, Tescan, Czech Republic) was utilized to observe the micro-/nanostructures. The elemental composition and map of the samples were determined by an energy-dispersive spectroscope (EDS, Tescan, Czech Republic). The three-dimensional morphology and cross-sectional profiles were characterized by a laser confocal microscope (LCM; Axio LSM700, Zeiss, Germany). The surface temperature distribution was recorded by an infrared camera (Ti450, Fluke, USA). A contact angle system (Biolin Scientific, Finland) was used to measure the contact angles of water droplets on the samples surfaces. The left and right contact angles were measured to calculate the average value of water contact angles (WCAs). All average WCAs and standard deviations were calculated from at least three different measurements.

## Results and Discussion

**Fig. 1:**
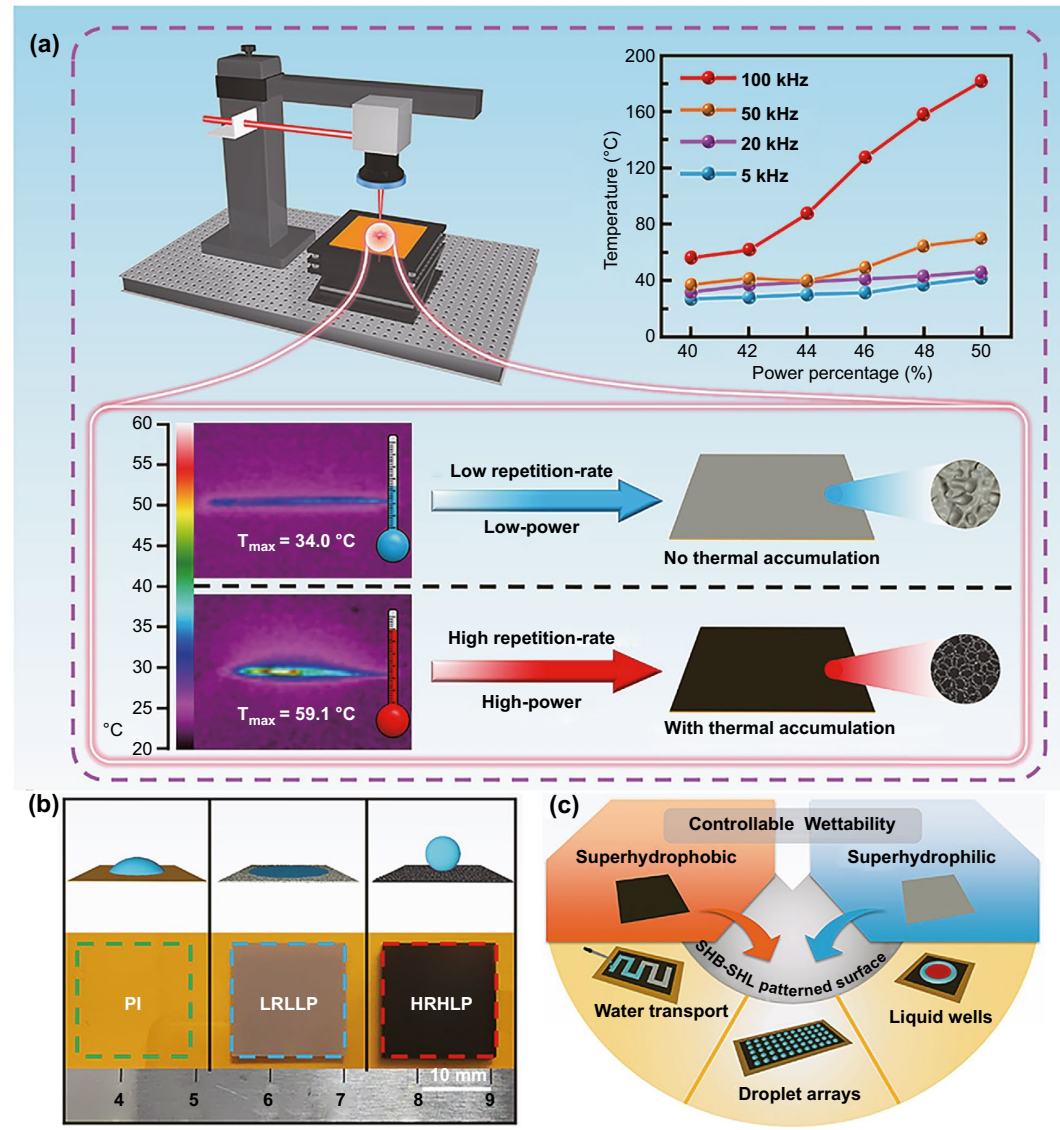
**a** Schematic diagram of the preparation of the laser-treated PI film. Maximum temperatures of treated PI film with different repetition rates and power percentages are shown on the upper right corner. **b** Optical photographs for pristine PI film, two types of laser-treated PI films and their surface wettability. **c** Various potential applications of superhydrophilic–superhydrophobic patterned surfaces

Femtosecond laser processing has drawn increasing attentions owing to its exceptional advantages, such as high resolution, non-contact processing, and strong controllability [40–45]. It can construct three-dimensional microstructures on different substrates and change the chemical composition of the substrates, making it as an effective technology for controlling the wettability of materials [46–53]. Figure 1a shows a schematic of the femtosecond laser-treated Polyimide (PI) film surface at different repetition rates and powers. In the case of low repetition rate (5 kHz) and low power (80 mW), worm-like rough microstructures were formed on the flat PI film surface by line-by-line scanning process, and the maximum temperature of the scanning process was ~ 34.0 °C. By comparison, under high repetition rate (100 kHz) and high power (900 mW), smooth PI film was transformed into rough film with grid-shaped microstructures. Correspondingly, the maximum temperature of this treatment was ~ 59.4 °C, which was significantly higher than that of low repetition rate and low power treatment. The maximum temperature of the femtosecond laser scanning process rose significantly with the increase in power percentage when the repetition rate reached or higher than 100 kHz, reflecting obvious thermal accumulation effects. Moreover, low thermal conductivity of PI film also promoted the local thermal accumulation effects. Generally, strong thermal accumulation effects induced by high repetition rate and high power can generate a larger thermal-affected region on the sample surface (Fig. S1) [54]. Figure 1b shows a photograph of the pristine PI film, low repetition rate and low power laser-treated Polyimide (LRLLP) film, high repetition rate and high power laser-treated Polyimide (HRHLP) film (Fig. S2). These three samples are hydrophilic, superhydrophilic, and superhydrophobic, respectively. A number of studies have investigated the wettability of PI treated with laser under various conditions (Table S1). Compared with the previous approaches for preparing surfaces with heterogeneous wettability, femtosecond laser thermal accumulation engineering can achieve controllable wettability by only one step in air atmosphere on PI film (Table S2). Owing to controllable wettability and designable patterns, superhydrophobic–superhydrophilic patterned surfaces (SHB-SHL) fabricated by the femtosecond laser thermal accumulation engineering have guaranteed the various applications of water transport, droplet arrays and liquid wells (Fig. 1c).

**Fig. 2:**
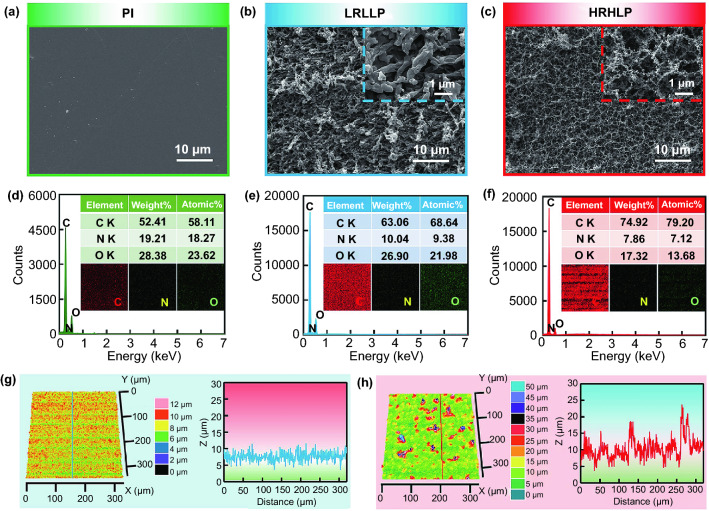
SEM images of **a** PI, **b** LRLLP, and **c** HRHLP film surfaces. Elemental chemical composition and maps of C, N, and O for **d** PI, **e** LRLLP, and **f** HRHLP film surfaces. 3D morphology and cross-sectional profiles for **g** LRLLP, and **h** HRHLP film surfaces

Figure 2a–c shows the scanning electron microscopy (SEM) images of the pristine and laser-treated PI film surfaces. Compared with the smooth surface of PI film (Fig. 2a), the LRLLP (5 kHz, 80 mW, 50 mm s^−1^) film is rough and covered by worm-like microstructures with an average width size of ~ 0.5 μm (Fig. 2b), the rough structures change the film surface wettability from hydrophilicity to superhydrophilicity. However, the HRHLP (100 kHz, 900 mW, 50 mm s^−1^) film surface is covered by grid-shaped microstructures composed of filaments covered with particles (Fig. 2c), which may be caused by strong thermal accumulation effects during femtosecond laser processing. The SEM images of different locations show that the rough microstructures are uniformly distributed on the laser-treated surfaces (Figs. S3 and S4). Additionally, SEM images of the treated PI film with laser scanning speeds of 50, 100, and 150 mm s^−1^ are shown in Figs. S5 and S6. The energy-dispersive spectroscope (EDS) was employed to determine the elemental chemical composition and maps of the PI, LRLLP and HRHLP films (Fig. 2d–f). The LRLLP film shows the increase in the C content from 58.11 to 68.64% and a decrease in N content, revealing that it was slightly carbonized (Fig. 2d-e). For the HRHLP film, the C content increased apparently from 58.11 to 79.20%, and the O content decreased from 23.62 to 13.68%, which suggested that the HRHLP film was seriously carbonized (Fig. 2d, f). The elemental maps indicated that C, N, and O were evenly distributed on PI films after the laser treatment. As can be seen from Fig. 2g, the uniformly morphology microstructures with a depth less than 5 μm were detected on the LRLLP film surface. Nevertheless, an LCM image of the HRHLP film surface shows protrusions with heights of 5–20 μm (Fig. 2h). Moreover, the LCM images of the treated PI film surface with laser scanning speeds of 50, 100, and 150 mm s^−1^ are also shown in Figs. S7 and S8.

**Fig. 3:**
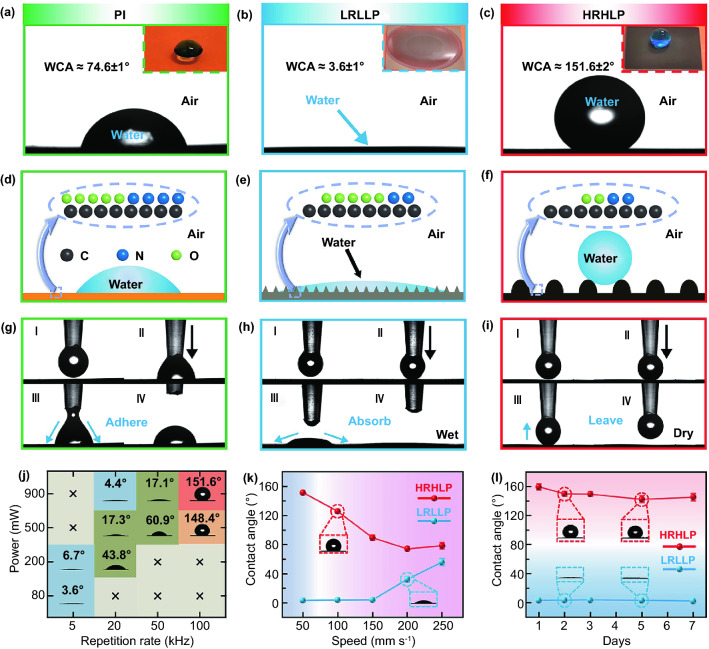
Static WCAs of **a** PI, **b** LRLLP, and **c** HRHLP films. Mechanism illustration of **d** PI, **e** LRLLP, and f HRHLP film surfaces wettability. Dynamic wetting behaviors of a water droplet (4 μL) on **g** PI, h LRLLP, and **i** HRHLP film surfaces. **j** Static WCA of the laser-treated PI film at different laser repetition rate and power. “ × ” means that the laser power cannot reach the designative value or is less than the damage threshold under the corresponding repetition rate. **k** WCAs of the LRLLP and HRHLP films with different laser scanning speeds. **l** WCAs of the LRLLP and HRHLP films placed in air for 7 days

Systematically investigating the surfaces wettability is of great importance. Water contact angle (WCA) measurements were, respectively, employed on the PI, LRLLP, HRHLP films surfaces to examine the water wettability. As shown in Fig. 3a, the PI film surface showed hydrophilicity with a WCA of ~ 74.6°, while the LRLLP film surface showed superhydrophilicity with a WCA of ~ 3.6° (Fig. 3b). Meanwhile, a small water droplet placed on the HRHLP film surface could remain spherically shaped, and the WCA reached ~ 151.6°, which indicated that the HRHLP film was superhydrophobic (Fig. 3c). Moreover, the HRHLP film surface exhibited a small sliding angle (~ 3°) and splendid self-cleaning effect (Figs. S9 and S10, Videos S1 and S2). The WCA only changed slightly when the substrate temperature increased from 30 to 70 °C or bending the HRHLP film for 30 cycles, revealing good thermal stability and resisting bending of the HRHLP film (Figs. S11 and S12). The LRLLP and HRHLP films also possessed superhydrophilicity and superhydrophobicity for other aqueous liquids, such as tea and coffee, respectively (Fig. S13). PI film surface is hydrophilic with a WCA of ~ 74.6°. After low repetition and low power laser treatment, the prepared sample surface is rough and covered by worm-like microstructures. In this condition, the water droplet could wet the microstructure of the rough surface. At this point, Wenzel state is used to understand superhydrophilicity:

1 $$\cos \theta_{W} = r\cos \theta_{Y}$$

where *r* is the surface roughness factor defined as the ratio of the actual contact area to projected area. *θ*_W_ and *θ*_Y_ are WCAs on rough and flat surfaces, respectively. This model and equation indicate that the surface roughness factor increases surface wettability for hydrophilic surfaces. Therefore, there is a smaller WCA (~ 3.6°) of the LRLLP film surface as compared to PI film surface. As such, the water droplet tends to spread quickly over the LRLLP film surface. By virtue of the amplifying function of the rough microstructures in surface wettability, compared with the hydrophilic PI film (Fig. 3d), the rough worm-like microstructures induced by low repetition rate laser ablation confer superhydrophilic property (Fig. 3e). However, SEM and LCM images of the HRHLP film surface show the presence of uniformly distributed micro-protrusions, causing the reduction in effective contact area of the HRHLP film surface in contact with water (Figs. 2h and S14). In general, a decrease in the O content on organic polymer surface attenuates surface hydrophilicity [55]. Besides surface topography, high repetition rate pulses can also cause a decrease in the O content from 23.62 to 13.68%, which has a great influence on surface wettability. In this situation, the water droplet could not completely wet the rough surface, and air pockets exist between the surface and water droplet. To understand the superhydrophobicity, the Cassie-Baxter model and Eq. (2) are used:

2 $$\cos \theta_{CB} = r_{f} *f\cos \theta_{Y} + f - 1$$

where *θ*_CB_ is the actual WCA, *r*_f_ is the ratio between the wet area and the apparent area, and *f* is the fraction of the rough surface in contact with water. The uniformly distributed micro-protrusions on the HRHLP film surface cause more air pockets and a low *f*, resulting in a larger WCA (~ 151.6°). Therefore, the synergistic function of chemical composition and surface microstructure endows the HRHLP film surface with superhydrophobic property (Fig. 3f).

Dynamic wetting behaviors of water droplets on the PI, LRLLP and HRHLP film surfaces were in accordance with their WCAs. When a water droplet from a microsyringe slowly contacted the PI film surface, it adhered to the PI film surface due to the high adhesion (Fig. 3g). When a water droplet contacted the LRLLP film surface, the water drop would be quickly absorbed and wet the whole LRLLP surface (Fig. 3h). However, when a water droplet touched the HRHLP film surface, the water droplet was extruded and deformed. Finally, it will leave the HRHLP surface with the gradual movement of the microsyringe, and the HRHLP surface remained dry, indicating a critically low adhesion of the HRHLP film surface (Fig. 3i). The videos of water droplets impact on the PI, LRLLP and HRHLP films surfaces were captured by a high-speed camera (Fig. S15, Videos S3-S5). In order to achieve controllable wettability, we tested the influence of the repetition rate, power and scanning speed on the wettability of the as-prepared sample. Figure 3j shows the influence of repetition rate and power on the surface wettability given a constant scanning speed (50 mm s^−1^). With negligible thermal accumulation, the samples treated with low repetition rate laser showed superhydrophilicity. The WCA of as-prepared samples tended to rise with the increase in repetition rate and laser power, which eventually achieved superhydrophobicity. When the laser power is constant, different repetition rates would cause completely different wetting ability. For example, in the case of power about 900 mW, repetition rates of 20, 50, and 100 kHz would induce superhydrophilicity, hydrophilicity, and superhydrophobicity, respectively. While, the change of power cannot produce controllable wettability under the same repetition rate. For instance, in the case of repetition rate about 100 kHz, powers of 200, 500, and 900 mW would induce hydrophilicity, hydrophilicity, and superhydrophilicity, respectively. It demonstrates the possibility of realizing controllable wettability by adjusting power and repetition rate under the condition of scanning speed about 50 mm s^−1^. As shown in Fig. 3k, once other parameters were certain, the series LRLLP and HRHLP films wettability would gradually approach the PI films with the scanning speed increase. Figure 3k indicates that the scanning speed of 50 mm s^−1^ is proper to achieve the superwettability. As can be seen from parts *j* and *k* of Fig. 3, the main parameter to control the surface wettability is the repetition rate, followed by power and scanning speed. In addition, the wettability performance of LRLLP and HRHLP films placed in air (temperature of ~ 20 °C and humidity of ~ 40%) for 7 days did not change significantly, demonstrating the excellent stability of LRLLP and HRHLP films (Fig. 3l). Therefore, it can be concluded that fabricating controllable wettability surfaces on PI film through femtosecond laser thermal accumulation engineering is feasible.

**Fig. 4:**
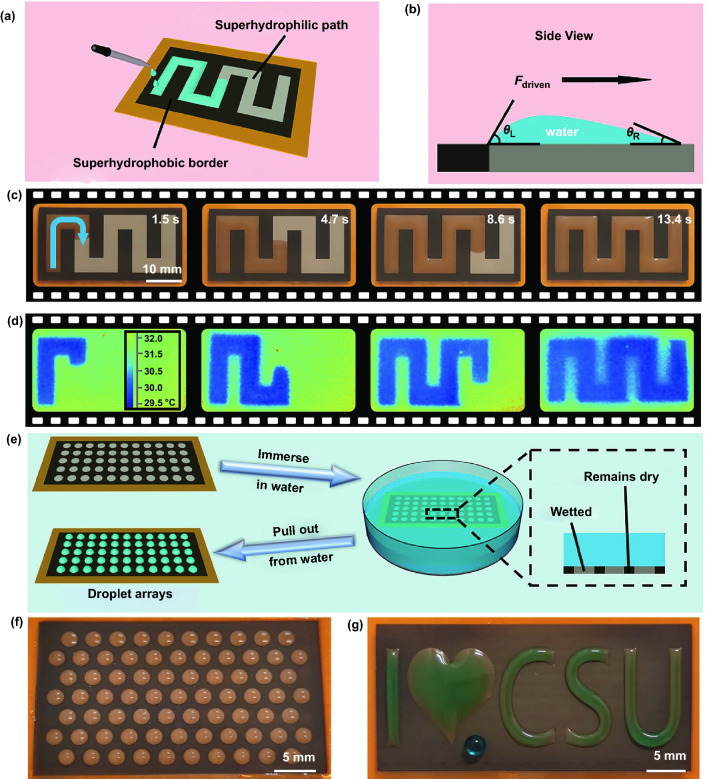
**a** Schematic diagram of water transport on superhydrophilic path. **b** Qualitative mechanism of water transport on superhydrophilic path. **c** A series of optical photographs for water transport along superhydrophilic path. **d** Infrared images of water transport along superhydrophilic path. **e** Schematic diagram and mechanism illustration of the constructed process for the droplet arrays. **f** Optical photographs for the droplet arrays. **g** Optical photographs for the designed superhydrophilic–superhydrophobic patterns filled with water dyed with Methylene Blue

By regulating the femtosecond laser thermal accumulation on different regions, various superhydrophilic–superhydrophobic patterns can be constructed, which realizes a series of new liquid manipulating applications. For example, path patterns composed of superhydrophobic borders and superhydrophilic paths could be used for continuous and stable liquids transportation. Figure 4a shows a schematic diagram of water transport. When water droplets continuously drop on one side of the superhydrophilic path, they speedily spread out and arrive at the other side. Figure 4b exhibits the qualitative mechanism of the water transport on the superhydrophobic–superhydrophilic path. Superhydrophobic borders ensure the uneven distribution of water to cause different transient angles. Thus, the difference in transient angles creates a driven force to make water transport along the designed superhydrophilic path, which can be described as follows [56, 57]:

3 $$F_{driven} \sim \gamma R_{0} \left( {\cos \theta_{R} - \cos \theta_{L} } \right)$$

where *θ*_R_ is the transient angle contacting with superhydrophilic paths, θ_L_ is the transient angle contacting with superhydrophobic borders, *R*_0_ is the characteristic radius of water droplets, and γ is the surface tension of water. Water-repellency from superhydrophobic borders and water-absorption from superhydrophilic paths ensure that water can transport along the designed superhydrophilic path. Figure 4c shows a series of optical photos of the whole process for water transport (Video S6). The trajectory of water transporting along the superhydrophilic path can also be seen from the corresponding infrared images (Fig. 4d). When encountering external force interference, the water on the superhydrophilic path can generate a rapid and adaptive deformation accordingly. This feature leads to the fact that when we tilted the substrate at a small angle in any direction, water could be limited to the superhydrophilic path (Video S7). With the gradual and continuous addition of water droplets, water was still kept on the superhydrophilic regions by forming a three-dimensional structure (Fig. S16).

Besides, uniformly distributed circular superhydrophilic areas surrounded by superhydrophobic regions were fabricated for creating droplet arrays (Video S8). As shown in Fig. 4e, the fabricated pattern was immersed in water and then pulled out from water. The optical image shows that water droplets were firmly adhered to superhydrophilic areas, but superhydrophobic regions remained dry (Fig. 4f). Using programable processing, a variety of patterns with different shapes were prepared, including triangle, rectangle, and hexagon, which could also form stable and consistent droplet arrays (Fig. S17). The droplet arrays composed of completely independent droplet units served as an ideal platform for high-throughput live-cell screenings without cross-contamination, which would be useful for various biological and medical applications. Additionally, water droplets were fixed on the designed superhydrophilic areas to generate various water-based patterns (Fig. 4g).

**Fig. 5:**
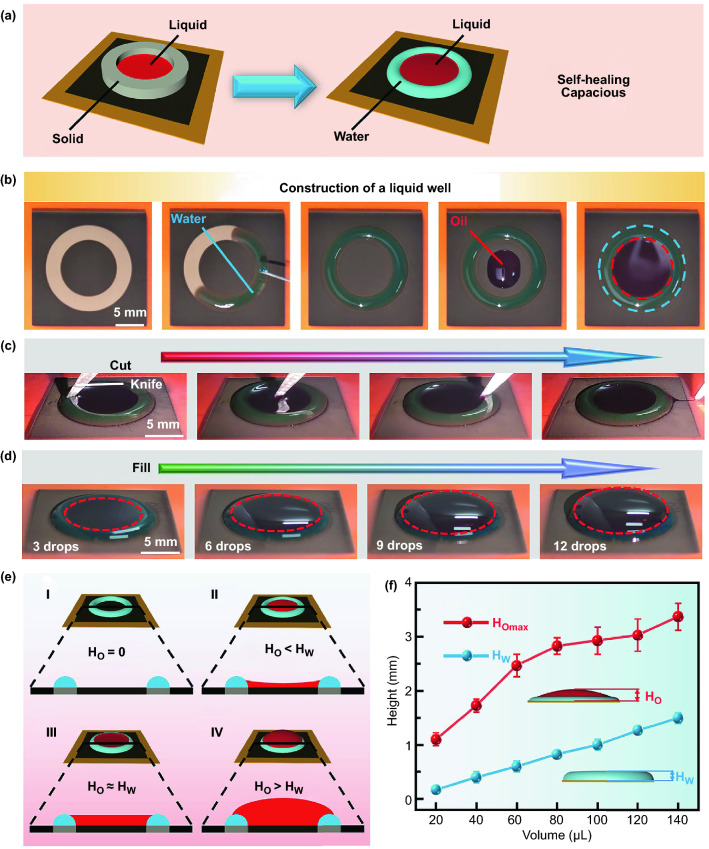
**a** Scheme diagram of a liquid contained in a solid vessel and inside a liquid well. **b** Optical photographs for the construction of a circular liquid well. Water constructed an annular water wall on the superhydrophilic areas. Oil was then added into the water wall. The inner and outer diameters of the superhydrophilic areas were 10 and 15 mm, respectively. **c** The self-healing properties of the liquid well using a knife to cut the liquid well. **d** A series of optical photographs for the liquid well with the increase in oil. **e** Scheme diagram for a cross section of the liquid well with different volumes of contained oil. **f** Maximum heights of the contained oil and height of an annular water wall as functions of water volume. All the used water and oil were dyed with Methylene Blue and Oil Red, respectively

Based on the water-repellency from superhydrophobic regions and the water-absorbing properties from superhydrophilic regions, water can be tethered to superhydrophilic areas surrounded by superhydrophobic regions to form stable water walls, which can contain another immiscible liquid. Compared with the traditional solid vessels, liquid wells have unique and intriguing features such as self-healing and capacious (Fig. 5a). In this experiment, oil was mainly selected as the contained liquid. Water was firstly confined to a superhydrophilic area to construct a water wall. Then, oil was added to the superhydrophobic regions surrounded by the water wall and spread out. Finally, oil was limited to the preformed water wall with an annular shape (Fig. 5b and Video S9). The results indicated that whether the organic liquids could be contained within the water walls mainly depends on the physical and chemical properties of the organic liquids (Fig. S18). Oil and 1-decanol incompatible with water could be well contained within the water wall. However, ethanol and isopropanol miscible with water collapsed the water wall and then spread out over the superhydrophobic regions. Combining with controllable femtosecond laser processing, we designed several patterns with different shapes (triangle, hexagon and conjoined square), which can contain 1-decanol successfully (Fig. S19). Besides, we employed a knife to cut the liquid well for testing its resistance to external mechanical forces. After cutting the liquid well filled with oil using a knife, the liquid well structure remained intact. Only a small part of oil droplets flowed out to the superhydrophobic regions surrounding the liquid well following the direction of the knife (Fig. 5c and Video S10).

Due to the dynamic fluidity of water, liquid wells could hold more organic liquid, which is completely distinct from the frequently used solid vessels. To investigate the high-capacity properties, oil was deposited on the superhydrophobic regions within a water wall (140 µL), and the volume of the oil was gradually increased until oil overflowed (Fig. 5d and Video S11). With the continuous addition of oil droplets, the water wall could accommodate a great deal of oil through an adaptive deformation. When less oil was deposited (Ho < Hw), oil menisci was formed in the area where the water wall contacted oil due to the tension between water and oil (Fig. 5e). When more oil was deposited (Ho > Hw), oil menisci was higher than the water wall, which is realized by the balance between capillary forces and gravitational forces on the top of the water wall (Fig. 5e). In addition, we inclined the liquid well with approximately the same height of oil and water wall at a small angle, then restored it to the horizontal surfaces (Fig. S20). During this process, oil was always successfully contained in the water wall due to the fluidity and deformation of water in the superhydrophilic areas. To further explore the capacity of a liquid well, the height of the water walls constructed by different water volumes and the maximum height of oil was measured. The results showed that a maximum height of 1.5 ± 0.1 mm was reached for 140 µL water. With a liquid wall built by 140 µL water, the maximum height of oil contained in the liquid well could reach 3.37 ± 0.25 mm (Fig. 5f). In the experimental cases, the maximum height of oil exceeded the corresponding water wall height, which reflected the high-capacity characteristics of liquid wells.

## Conclusions

In summary, we processed patternable and controllable liquid manipulating surfaces on PI films through one-step femtosecond laser thermal accumulation engineering. With various surface microstructures and chemical composition induced by thermal accumulation effects, the laser-treated samples realized continuously controllable wettability from superhydrophilicity (~ 3.6°) to superhydrophobicity (~ 151.6°). By regulating local thermal accumulation effects, various surfaces with patternable wetting performance were also successfully fabricated and multiple liquid manipulating applications were achieved, such as water transport, droplet arrays, and liquid wells. The proposed facile and efficient fabrication method might provide a viable source for various applications of bubble self-assembling, droplet-solid impacting behavior, fog collection, and oil–water separation.

## Supplementary Information

Below is the link to the electronic supplementary material.

Supplementary file1 (PDF 2249 KB)

Supplementary file2 (AVI 1643 KB)

Supplementary file3 (AVI 2392 KB)

Supplementary file4 (AVI 1428 KB)

Supplementary file5 (AVI 1538 KB)

Supplementary file6 (AVI 1679 KB)

Supplementary file7 (AVI 1526 KB)

Supplementary file8 (AVI 3750 KB)

Supplementary file9 (AVI 3180 KB)

Supplementary file10 (AVI 1605 KB)

Supplementary file11 (AVI 604 KB)

Supplementary file12 (AVI 7806 KB)
