# Supplementary material for: Femtosecond Laser Thermal Accumulation-Triggered Micro-/Nanostructures with Patternable and Controllable Wettability Towards Liquid Manipulating
Source: Nanomicro Lett. 2022 Apr 8;14:97. doi: 10.1007/s40820-022-00840-6 (PMC8993985; doi:10.1007/s40820-022-00840-6)
Supplement: Supplementary file 1 — Supplementary file1 (PDF 2249 KB) [file 40820_2022_840_MOESM1_ESM.pdf]

Supporting Information for

Femtosecond Laser Thermal Accumulation Triggered Micro/Nanostructures with Patternable and Controllable Wettability Towards Liquid Manipulating

Kai Yin[1, 2], Lingxiao Wang[1], Qinwen Deng[1], Qiaoqiao Huang[1], Jie Jiang[1, *], Guoqiang Li [3, *], Jun He [1, *]

[1]Hunan Key Laboratory of Nanophotonics and Devices, School of Physics and Electronics, Central South University, Changsha 410083, P. R. China

[2]The State Key Laboratory of High Performance and Complex Manufacturing, College of Mechanical and Electrical Engineering, Central South University, Changsha 410083, P. R. China

[3]Key Laboratory of Testing Technology for Manufacturing Process of Ministry of Education, Southwest University of Science and Technology, Mianyang 621010, P. R. China

*Corresponding authors. E-mail: jiangjie@csu.edu.cn (J. Jiang), guoqli@swust.edu.cn (G. Li), junhe@csu.edu.cn (J. He)

Supplementary Figures and Tables

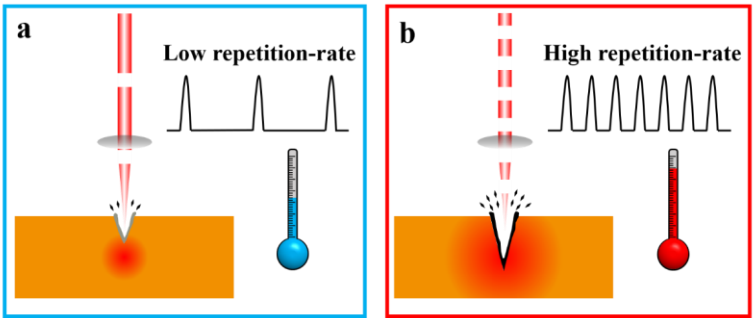

Fig. S1 Schematic diagram of **a** low repetition rate, and **b** high repetition rate laser treated PI film in air

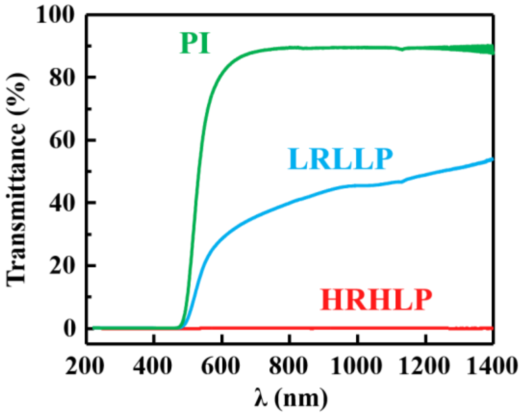

Fig. S2 Transmittance of the PI, LRLLP and HRHLP films in the spectral range of 220-1400 nm

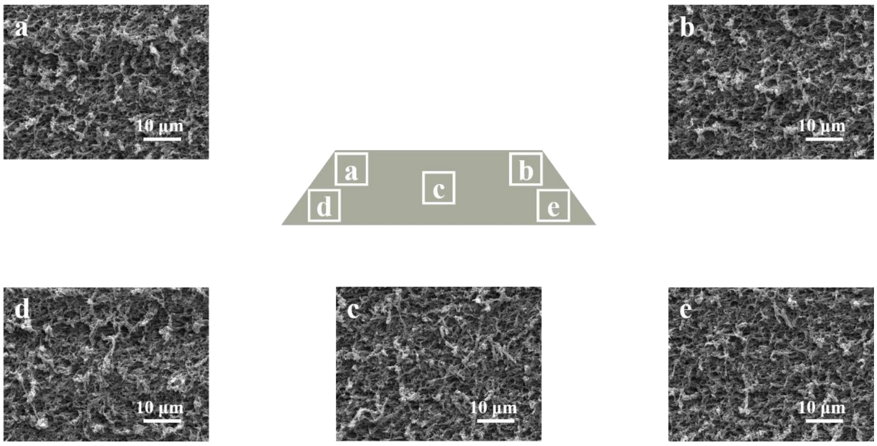

Fig. S3 SEM images of various positions of the LRLLP film surface

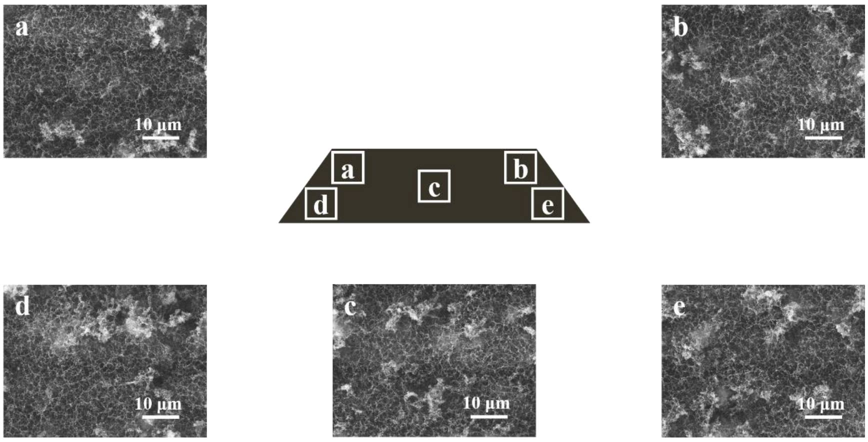

Fig. S4 SEM images of various positions of the HRHLP film surface

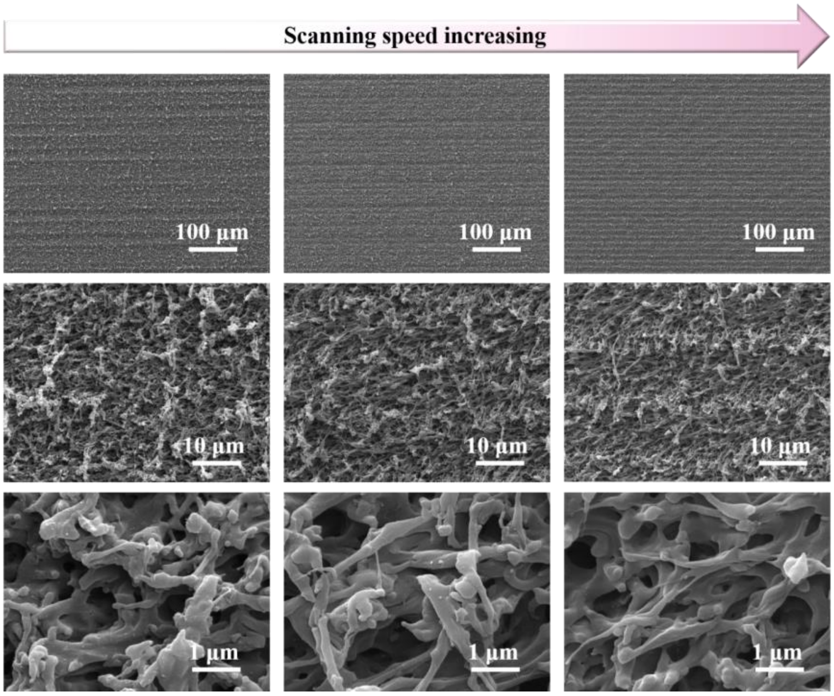

Fig. S5 SEM images of LRLLP (5 kHz, 80 mW) film surfaces with the scanning speeds of 50, 100, and 150 mm s^{-1}. The downsets are corresponding magnified images

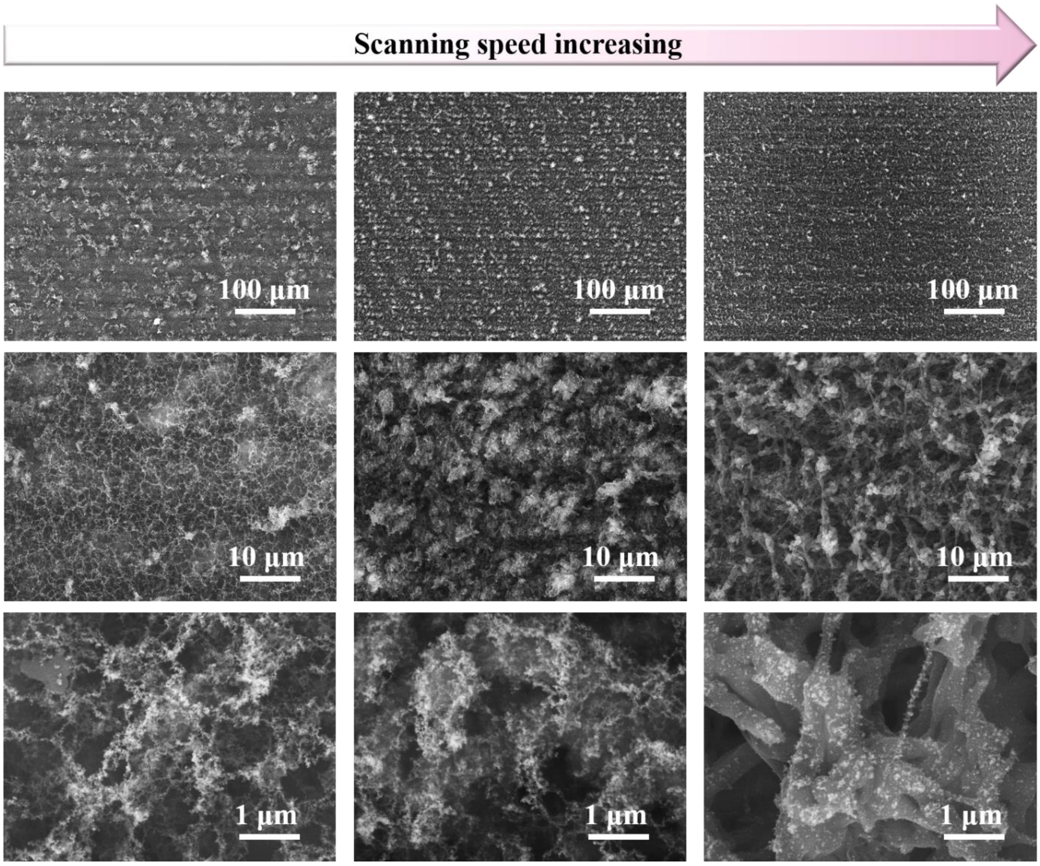

Fig. S6 SEM images of HRHLP (100 kHz, 900 mW) film surfaces with the scanning speeds of 50, 100, and 150 mm s^{-1}, respectively. The downsets are corresponding magnified images

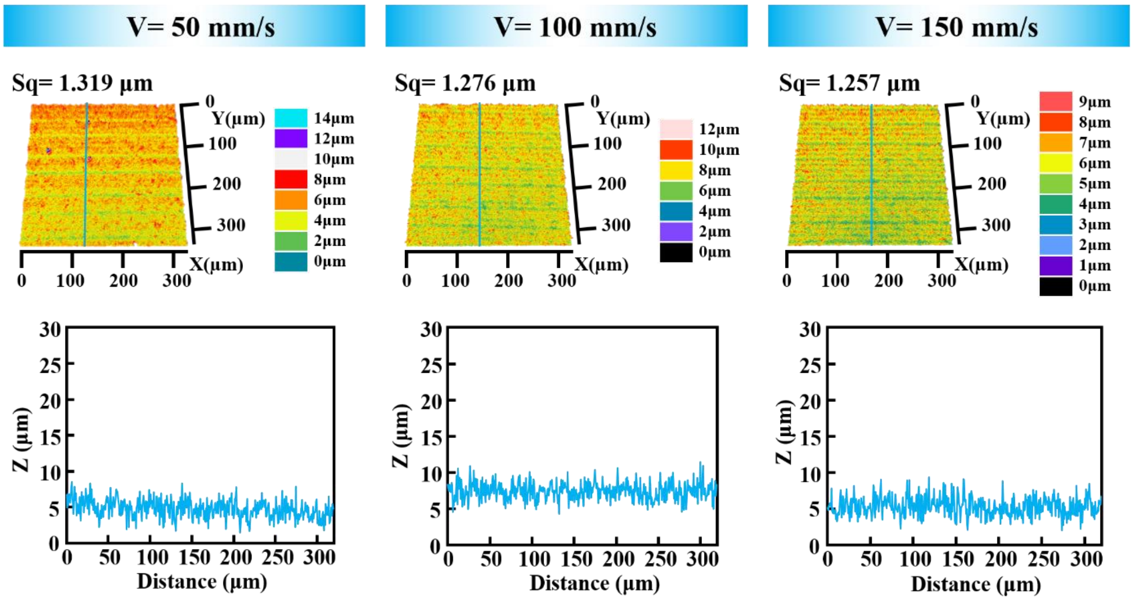

Fig. S7 LCM images of LRLLP (5 kHz, 80 mW) film surfaces with the scanning speeds of 50, 100, and 150 mm s^{-1}

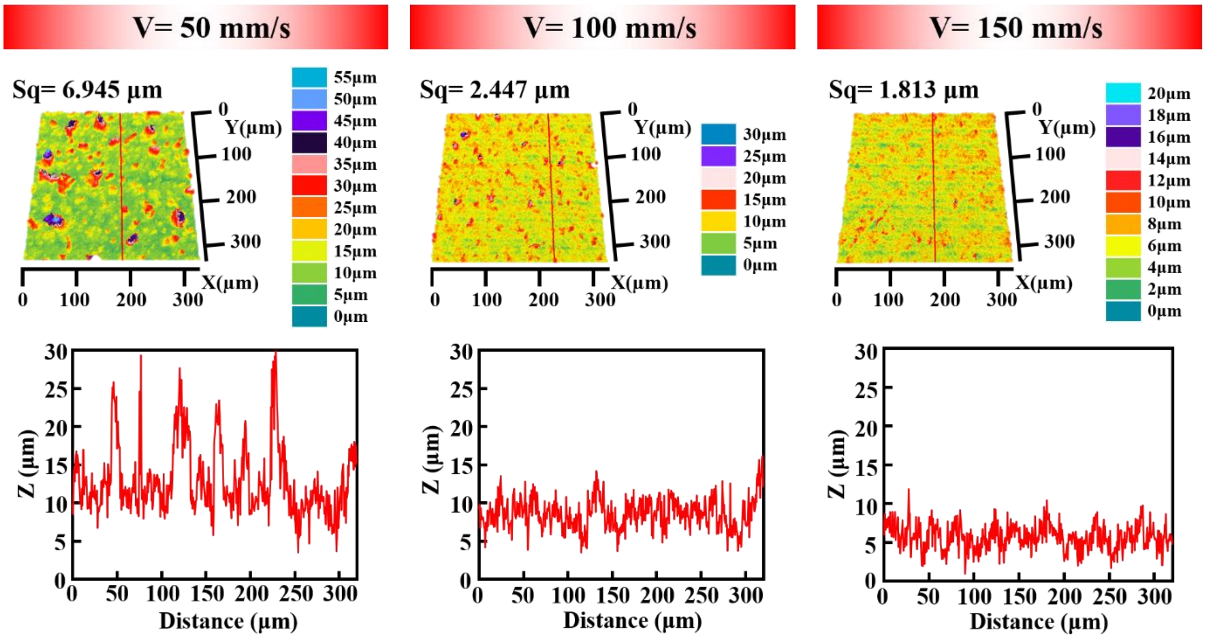

Fig. S8 LCM images of HRHLP (100 kHz, 900 mW) film surfaces with the scanning speeds of 50, 100, and 150 mm s^{-1}

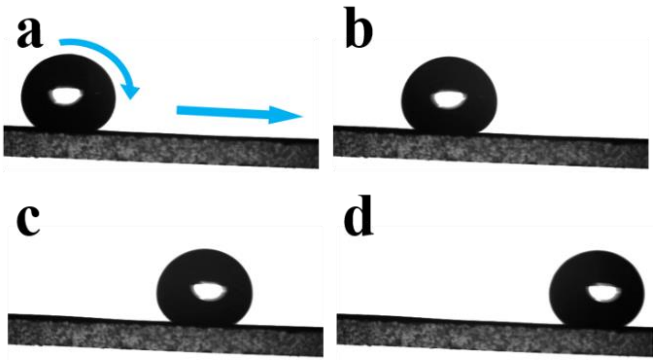

Fig. S9 Pictures of a water droplet sliding (~3 °) on the HRHLP film surface

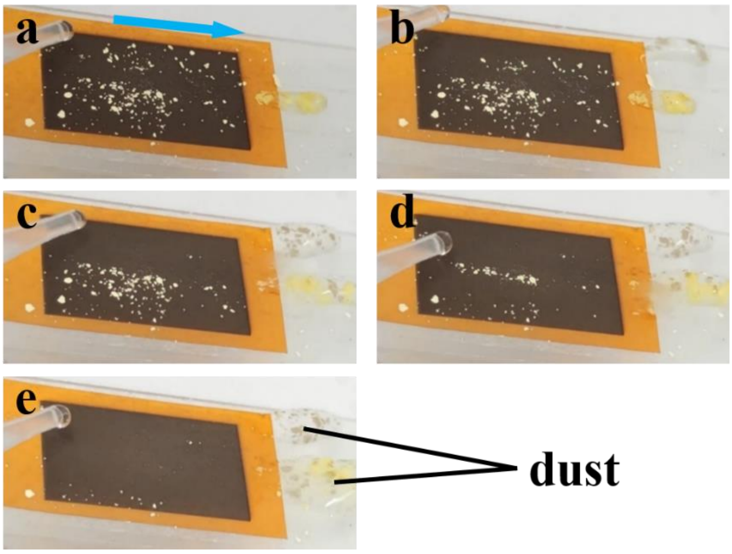

Fig. S10 Self-cleaning demonstration of the HRHLP film

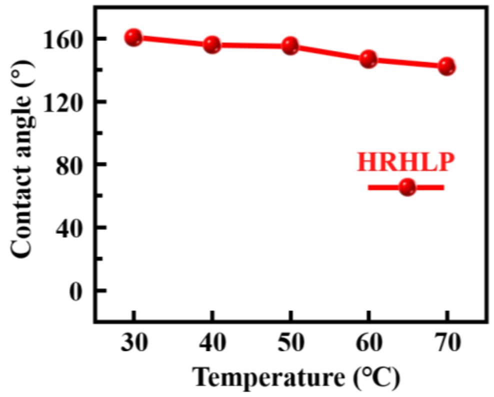

Fig. S11 Thermal stability test of the HRHLP film wettability

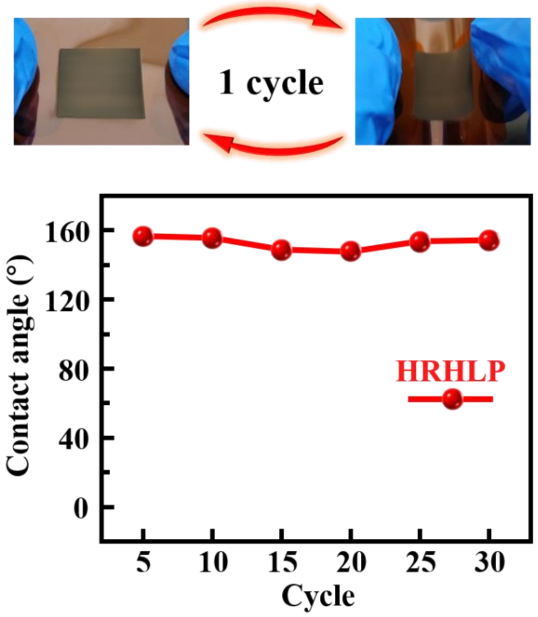

Fig. S12 Resisting bending test for the HRHLP film wettability

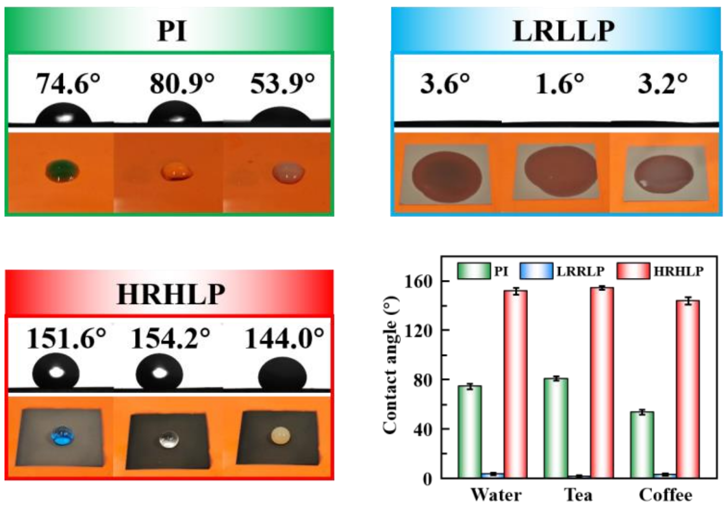

Fig. S13 Three liquids (water, tea and coffee) placed on the PI, LRLLP and HRHLP films and comparison of contact angles. The used water was dyed with Methylene Blue

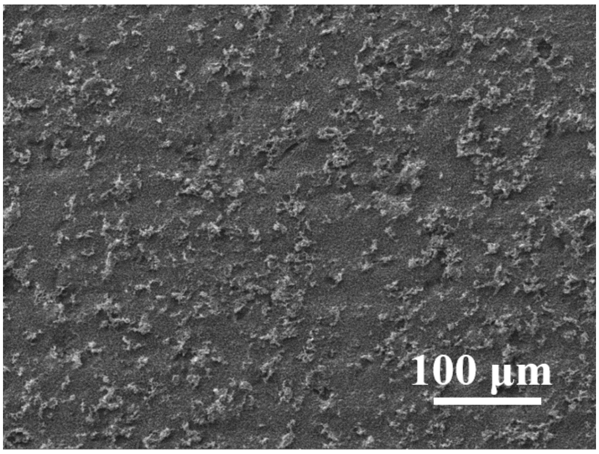

Fig. S14 SEM images of HRHLP (100 kHz, 900 mW, 50 mm s^{-1}) film surface, indicating the presence of uniformly distributed micro-protrusions

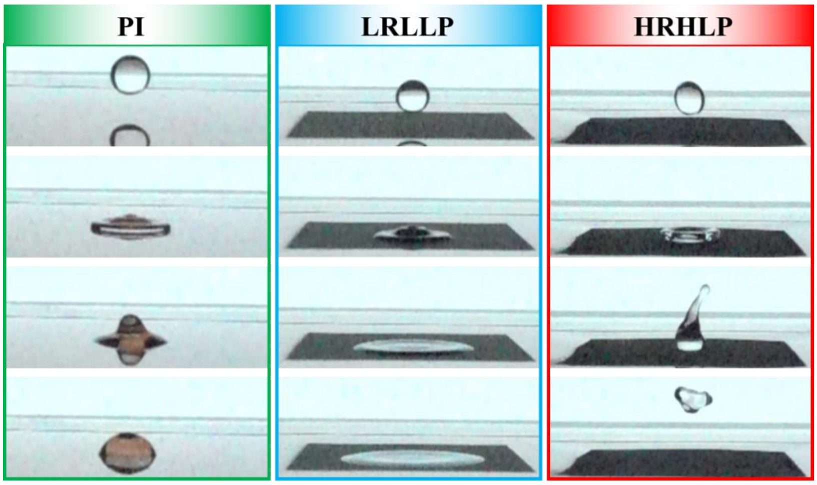

Fig. S15 Sequential photographs of a water droplet impact on the PI, LRLLP and HRHLP film surfaces

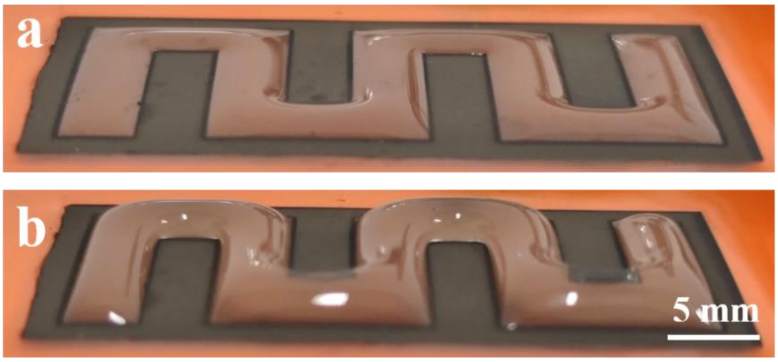

Fig. S16 Shape comparison of some and much water on the superhydrophilic paths

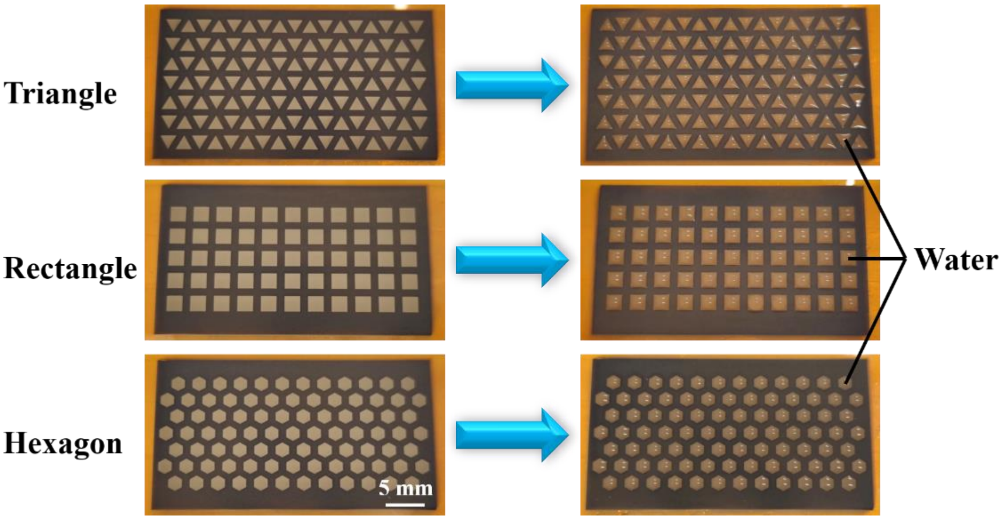

Fig. S17 Various patterns with different shapes (triangle, rectangle, and hexagon) to form droplet arrays

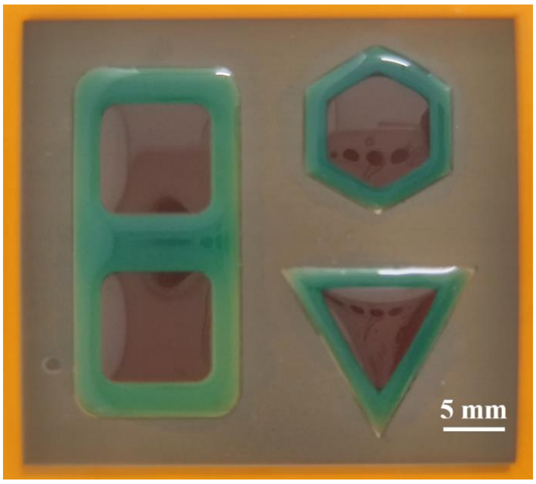

Fig. S18 Images of liquid wells with various shapes (triangle, hexagon and conjoined square) containing 1-decanol

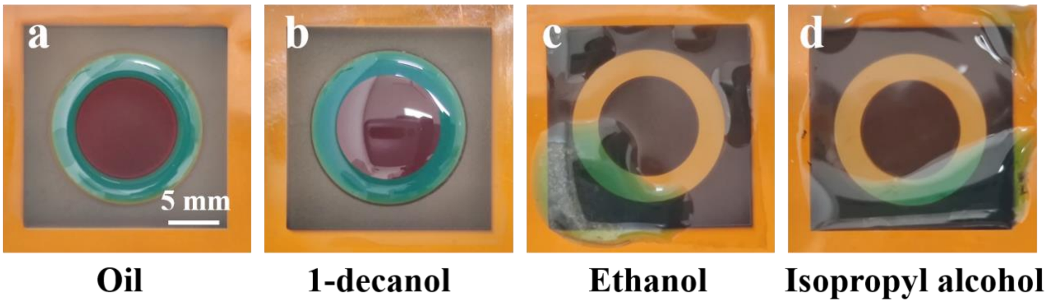

Fig. S19 Confinement for different organic liquids (red) with a water wall (blue)

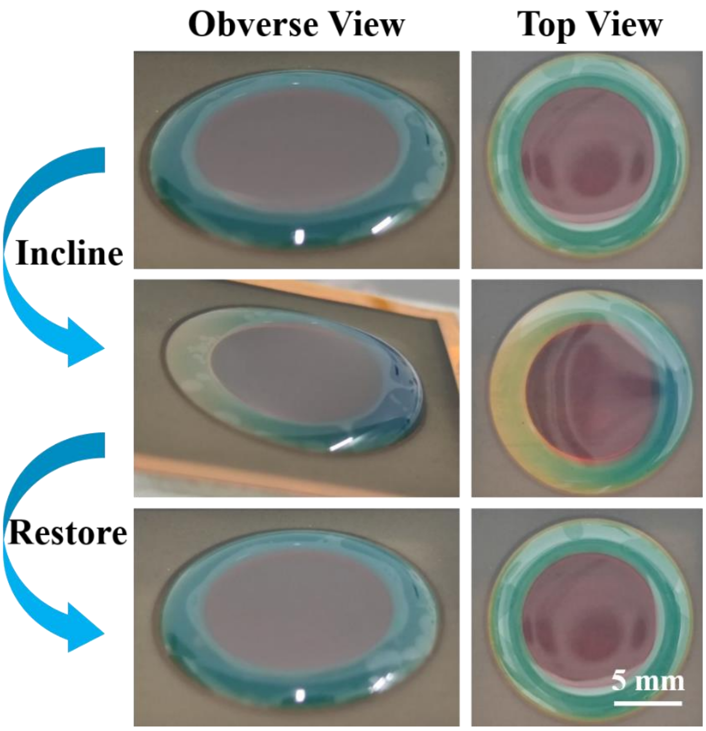

Fig. S20 Location stability test for the liquid well. The height of the contained oil is approximately equal to the water wall (60 μL)

Table S1 Wettability of the laser-treated PI surfaces under different conditions

Laser source	Addition	Atmosphere	Wettability	Post treatment	Ref.
Laser	None	Air	Superhydrophilic (~0 °)	O_2 plasma treatment	[1]
CW infrared CO_2 laser	$KMnO_4$	Air	Superhydrophilic (~0 °)	None	[2]
Universal laser systems	None	Ar	Superhydrophobic (~152 °)	None	[3]
1060 nm CO_2 laser	None	N_2	Hydrophobic (~140 °)	None	[4]
1064 nm Nd:YAG laser	Gelatin	Air	Hydrophilic to superhydrophobic (24.3 °–153.5 °)	None	[5]
1060 nm CO_2 laser	None	Air	Hydrophobic (~131.3 °)	None	[6]
Femtosecond fiber laser	None	Air	Superhydrophilic to superhydrophobic (3.6 °–151.6 °)	None	Our work

Table S2 Different methods to construct surfaces with heterogeneous wettability

Main material	Main Method	Consuming step	Wettability	Post treatment	Ref.
Slide glass	Laser-induced backward transfer technique	Two steps	Superhydrophilic/ superhydrophobic	Fluoroalkylsilane ethanol solution	[7]
6061 Al	Electrochemical etching	Two steps	Superhydrophilic/ superhydrophobic	Fluoroalkylsilane ethanol solution	[8]
Aluminum	Inkjet-printing	Five steps	Superhydrophilic/ superhydrophobic	Perfluorodecyl trimethoxysilane	[9]
Stainless steel mesh	Spray-coating	Four steps	Superhydrophilic/ superhydrophobic	None	[10]
Aluminum	Nanosecond laser ablation	Three steps	Controllable	Boiling water and heat treatment	[11]
PE-coated paper	Spark-coating	Two steps	Superhydrophilic/ superhydrophobic	None	[12]
PI	Femtosecond laser thermal accumulation	One step	Controllable	None	Our work

Video S1 A water droplet sliding on the HRHLP film surface which is inclined about 3°.

Video S2 Self-cleaning effect examination of the HRHLP film. The chalk powder was picked up by the rolling water droplets and readily removed.

Video S3 A water droplet impacting on the PI film surface. The water droplet fell, spread, and finally adhered to PI film surface.

Video S4 A water droplet impacting on the LRLLP film surface. The water droplet fell and was quickly absorbed.

Video S5 A water droplet impacting on the HRHLP film surface. The water droplet experienced falling, spreading, retracting, and finally rebounding back into the air.

Video S6 The whole process for water transportation on the superhydrophilic path.

Video S7 Location stability test for the water limited to the superhydrophilic path surrounded by the superhydrophobic border.

Video S8 The process of creating droplet arrays through immersing the fabricated pattern in water and then pulling it out from water.

Video S9 Formation of a liquid well on a superhydrophobic-superhydrophilic patterned surface. The organic solvent is oil (dyed red).

Video S10 Cutting a liquid well with a knife. The liquid well structure remained intact after cutting by a knife.

Video S11 Capacity test for a liquid well. The water wall can contain amounts of oil through an adaptive deformation.

Supplementary References

[S1] D. Han, Z. Chen, J. Li, J. Mao, Z. Jiao et al., Airflow enhanced solar evaporation based Janus graphene membranes with stable interfacial floatability. ACS Appl. Mater. Interfaces **12**(22), 25435-25443 (2020). https://doi.org/10.1021/acsami.0c05401

[S2] J. Liu, H. Liu, N. Lin, Y. Xie, S. Bai et al., Facile fabrication of super-hydrophilic porous graphene with ultra-fast spreading feature and capillary effect by direct laser writing. Mater. Chem. Phys. **251**, 123083 (2020). https://doi.org/10.1016/j.matchemphys.2020.123083

[S3] Y. Li, D. Luong, J. Zhang, Y.R. Tarkunde, C. Kittrell et al., Laser-induced graphene in controlled atmopheres: from superhydrophilic to superhydrophobic surfaces. Adv. Mater. **29**(27), 1700496 (2017). http://dx.doi.org/10.1002/adma.201700496

[S4] L. Huang, M. Gu, Z. Wang, T. Tang, Z. Zhu et al., Highly efficient and rapid inactivation of coronavirus on non-metal hydrophobic laser-induced graphene in mild conditions. Adv. Funct. Mater. **31**(24), 2101195 (2021). https://doi.org/10.1002/adfm.202101195

[S5] W. Wang, L. Lu, Y. Xie, Z. Li, W. Wu et al., One-step laser induced conversion of a gelatin-coated polyimide film into graphene: tunable morphology, surface wettability and microsupercapacitor applications. Sci. China Technol. Sci. **64**, 1030-1040 (2021). https://doi.org/10.1007/s11431-020-1609-4

[S6] M. Kim, K. Yang, Y.S. Kim, J.C. Won, P. Kang et al., Laser-induced photothermal generation of flexible and salt-resistant monolithic bilayer membranes for efficient solar desalination. Carbon **164**, 349-356 (2020). https://doi.org/10.1016/j.carbon.2020.03.059

[S7] J. Zhang, F. Chen, Y. Lu, Z. Zhang, J. Liu et al., Superhydrophilic-superhydrophobic patterned surfaces on glass substrate for water harvesting. J. Mater. Sci. **55**, 498-508 (2020). https://doi.org/10.1007/s10853-019-04046-x

[S8] X. Yang, J. Song, J. Liu, X. Liu, Z. Jin, A twice electrochemical-etching method to fabricate superhydrophobic-superhydrophilic patterns for biomimetic fog harvest. Sci. Rep. **7**, 8816 (2017). https://doi.org/10.1038/s41598-017-09108-1

[S9] J. Sun, Y. Li, G. Liu, F. Chu, C. Chen et al., Patterning a superhydrophobic area on a facile fabricated superhydrophilic layer based on an inkjet-printed water-soluble polymer template. Langmuir **36**(33), 9952-9959 (2020). https://doi.org/10.1021/acs.langmuir.0c01769

[S10] J. Feng, L. Zhong, Z. Guo, Sprayed hieratical biomimetic superhydrophilic-superhydrophobic surface for efficient fog harvesting. Chem. Eng. J. **388**, 124283 (2020). https://doi.org/10.1016/j.cej.2020.124283

[S11] C.V. Ngo, D.M. Chun, Control of laser-ablated aluminum surface wettability to superhydrophobic or superhydrophilic through simple heat treatment or water boiling post-processing. Appl. Surf. Sci. **435**, 974-982 (2018). https://doi.org/10.1016/j.apsusc.2017.11.185

[S12] T. Kumpika, E. Kantarak, W. Sroila, A. Panthawan, N. Jhuntama et al., Superhydrophilic/superhydrophobic surfaces fabricated by spark-coating. Surf. Interface Anal. **50**(8), 827-834 (2018). https://doi.org/10.1002/sia.6485
